# Inter- and Intra-Species Diversity of Lactic Acid Bacteria in *Apis mellifera ligustica* Colonies

**DOI:** 10.3390/microorganisms8101578

**Published:** 2020-10-14

**Authors:** Massimo Iorizzo, Gianfranco Pannella, Silvia Jane Lombardi, Sonia Ganassi, Bruno Testa, Mariantonietta Succi, Elena Sorrentino, Sonia Petrarca, Antonio De Cristofaro, Raffaele Coppola, Patrizio Tremonte

**Affiliations:** 1 Department of Agricultural, Environmental and Food Sciences, University of Molise, 86100 Campobasso, Italy; iorizzo@unimol.it (M.I.); gianfranco.pannella@unimol.it (G.P.); sonia.ganassi@unimol.it (S.G.); bruno.testa@unimol.it (B.T.); succi@unimol.it (M.S.); sorrentino@unimol.it (E.S.); decrist@unimol.it (A.D.C.); coppola@unimol.it (R.C.); tremonte@unimol.it (P.T.); 2 Consorzio Nazionale Produttori Apistici CONAPROA, 86100 Campobasso, Italy; sonia_petrarca@libero.it

**Keywords:** honey bee foragers, gut microbiota, beebread, honey stomach, midgut

## Abstract

Lactic acid bacteria could positively affect the health of honey bees, including nutritional supplementation, immune system development and pathogen colonization resistance. Based on these considerations the present study evaluated predominant Lactic Acid Bacteria (LAB) species from beebread as well as from the social stomach and midgut of *Apis mellifera ligustica* honey bee foragers. In detail, for each compartment, the diversity in species and biotypes was ascertained through multiple culture-dependent approaches, consisting of Polymerase Chain Reaction-Denaturing Gradient Gel Electrophoresis (PCR-DGGE), 16S rRNA gene sequencing and Randomly Amplified Polymorphic DNA-Polymerase Chain Reaction (RAPD-PCR). The study of a lactic acid bacteria community, performed with PCR-DGGE and sequence analysis targeting the V1–V3 region of the 16S rRNA gene (rDNA), highlighted the presence of a few species, including *Apilactobacillus kunkeei*, *Lactiplantibacillus plantarum, Fructobacillus fructosus*, *Levilactobacillus brevis* and *Lactobacillus delbrueckii subsp. lactis*. Depending on the different compartments, diverse levels of biodiversity in species were found. Particularly, a very low inter-species biodiversity was detected in the midgut that was prevalently dominated by the presence of *Apilactobacillus kunkeei*. On the other hand, the beebread was characterized by a reasonable biodiversity showing the presence of five species and the predominance of *Apilactobacillus kunkeei*, *Lactiplantibacillus plantarum and Fructobacillus fructosus*. The RAPD-PCR analysis performed on the three predominant species allowed the differentiation into several biotypes for each species. Moreover, a relationship between biotypes and compartments has been detected and each biotype was able to express a specific biochemical profile. The biotypes that populated the social stomach and midgut were able to metabolize sugars considered toxic for bees while those isolated from beebread could contribute to release useful compounds with functional properties. Based on this knowledge, new biotechnological approaches could be developed to improve the health of honey bees and the quality of bee products.

## 1. Introduction

The gut microbial composition of honey bees (*Apis mellifera*) is simple, unique and highly specialized; the dominant bacterial phyla belong to the *Proteobacteria*, *Actinobacteria*, *Bacteroidetes* and *Firmicutes* [1,2]. In social insects, such as honey bees, intestinal bacteria are transmitted and shared by colony members through oral–faecal transmission, oral trophallactic interaction, consumption of stored pollen or beebread, interactions with older bees within the hive and contact with hive material during the adult phase [3,4]. With its simple and characteristic composition, the honey bee gut microbiota provides an excellent model for studying the functional and structural aspects of the bacterial communities associated with the gut [5,6,7].

These microorganisms participate in various processes such as food digestion, detoxification from harmful molecules, supply of essential nutrients, participation in the host defense system and protection from pathogens and parasites [3]. An imbalance of this microbiota (dysbiosis) can lead to a greater susceptibility to disease and an alteration of the genetic-immune system of bees. Dysbiosis can lead to a weakening of bees that are unable to cope with external stressors and pathogens and opportunistic diseases, contributing to the phenomenon of Colony Collapse Disorder (CCD) [6,8,9,10,11,12,13,14]. The presence of Lactic Acid Bacteria (LAB) in the honey bee digestive system has been consistently reported in the literature [15,16].

The environment of the honey bee stomach is characterized by the microaerobic condition, the presence of nectar sugars and sufficiently optimal temperature (35 °C), independent from the outside air temperature, and is the optimal niche for LAB. Based on the received data, Olofsson et al. [17] suggested that bees and LAB flora developed with a mutual dependence on each other: LAB received a niche in which nutrients were available, and the bees obtained a protection from harm [18,19,20]. The LAB originate from the flower nectar and/or pollen grains collected and ingested by the bees and are found in the gut, stored pollen and honey. The pollen collected from flowers changes both microbiologically and biochemically immediately after harvesting, thanks to the contribution of lactic acid bacteria that ferment sugars. The pollen is thus transformed into beebread to support the young growing bees that are the future of the colony [15,19,21,22,23]. Besides, the LAB microbiota has been linked to the immune system and defense against pathogens [24,25]. This has stimulated the search for greater knowledge on the relationship between the composition and function of the intestinal microbiota with the health and productivity of bees [8,26,27,28,29]. The ever-deeper knowledge of the LAB populations, which colonize the intestines of bees, will surely enrich our understanding on insect–microbe symbiosis and on the evolutionary dynamics of these microorganisms in the social life of the hive. *A. m. ligustica* is a native and an endemic Italian subspecies easily bred by beekeepers because of its adaptability to a wide range of climatic conditions, its ability to store large quantities of honey without swarming and its docility if disturbed.

In recent years, a major concern has arisen in several countries for the conservation of local honey bee species and of their original genetic structure [30]. The knowledge about LAB species diversity could be crucial for the development of biotechnological approaches able to improve the health of honey bees. On this basis, the aim of this study was the phenotypic and genotypic characterization of the predominant LAB species from the social stomach and from midgut of *A. m. ligustica* honey bee foragers and from the beebread produced in its hives. The research was carried out in Molise region (Italy), an area with a temperate climate in the Southern Italy.

## 2. Materials and Methods

### 2.1. Sample Collection

The samples of honey bee foragers and of beebread were collected from *A. m. ligustica* hives within apiaries sites in Mirabello Sannitico (41°30′59″56 N; 14°40′25″68 E), Spinete (41°32′39″24 N; 14°29′14″56 E), Busso (41°33′18″36 N; 14°33′42″84 E) and Pozzilli (41°30′44″64 N; 14°3′48″24 E), Molise Region (Italy), during the month of June 2019. At Mirabello Sannitico the apiary was formed by 25 colonies, at Spinete by 30, Busso by 20 and Pozzilli by 50. For each apiary three hives were selected and, for each hive, one sample formed five honey bee foragers which were collected and taken alive to the laboratory for dissection to obtain one sample of their midgut and one of honey stomach. The beebread, one sample (2 g) for hive, was taken using a sterile spatula, placed into sterile tubes and subjected to analysis on the same day.

### 2.2. Sample Processing

The LAB isolation was carried out by dissecting the social stomach and midgut of foragers and from the beebread. To dissect and separate the social stomach and midgut from bee samples, they were kept in the refrigerator for at least 5 min, devitalized by placing gentle pressure on the prothorax and then dissected, at room temperature, in a glass Petri dish containing sterile saline solution (9.0 g/L of NaCl). The dissection was carried out utilizing stainless-steel scissors for microdissection and a curved lancet (both washed in alcohol and sterilized with flame). The social stomach has been obtained by cutting the distal part of the esophagus and close to the proventriculus. The midgut was obtained by making a further incision at the level of the pyloric valve. The anatomical samples, of 5 foragers, were placed in the respective sterile glass tubes containing sterile saline solution for the subsequent isolation of the bacteria. Ten grams of each sample of beebread were placed in 90 mL of a physiological solution and serial decimal dilutions were obtained.

### 2.3. Bacterial Isolation

The social stomachs, the midguts and the beebread, in sterile saline solution were homogenized and serial decimal dilutions were obtained. Briefly, LAB were enumerated and isolated by plating serial decimal dilutions on MRS agar medium (Oxoid, Milan, Italy) adding 40 mg/L cycloheximide, on modified MRS containing 10% fructose. Plates were incubated for 48–72 h at 30 °C under anaerobic conditions using an anaerobic system (Anaerogen, Oxoid, Milan, Italy).

Representative numbers (10%) of colonies randomly picked from each assayed medium were purified by streaking on the suitable agar media. The colonies were randomly selected according to morphological differences (colony size and shape).

The pure isolates were tested for their Gram reaction, catalase activity and morphology. Gram-positive and catalase-negative were selected as presumptive LAB and were stored at − 80 °C in the corresponding liquid isolation medium, supplemented with 25% (*v/v*) of glycerol.

### 2.4. Genotypic Characterization

#### 2.4.1. DNA Extraction

Two milliliters of each overnight isolate cultures were centrifuged at 14,000× *g* for 10 min at 4 °C and the pellet obtained was subjected to DNA extraction according to Querol et al. [31], with the addition of lysozyme (25 mg/mL, Sigma-Aldrich, Milan, Italy) and mutanolysin (10 U/mL, Sigma-Aldrich) for bacterial cell-wall digestion. The quantity and purity of the DNA were assessed by an optical reading at 260 and 280 nm, as described by Sambrook et al. [32].

#### 2.4.2. Polymerase Chain Reaction-Denaturing Gradient Gel Electrophoresis (PCR-DGGE) Analysis

The DNA from each isolate was prepared for PCR analysis by amplifying the V1 region of 16S rRNA using as primers: P1V1 (5′-GCG GCG TGC CTA ATA CAT GC-3′) [33] and P2V1 (5′-TTC CCC ACG CGT TAC TCA CC-3′) [34]. A GC clamp (5′-CGC CCG CCG CGC CCC GCG CCC GTC CCG CCG CCC CCG CCC G-3′) [35] was attached to the 5′ end of the P1V1 primer. PCR was performed in a Mastercycler gradient (Eppendorf, Hamburg, Germany).

The reaction mixture (50 μL) consisted of 10 mmol/L Tris–HCl (pH 8.3), 50 mmol/L KCl, 200 μmol/L of each dATP, dGTP, dCTP and dTTP, 1.5 mmol/L MgCl, 0.2 μmol/L of each primer, 200 ng DNA and 1.25 U *Taq*-DNA polymerase (Thermo Fisher Scientific, Rodano, Milan, Italy). The amplification program consisted of a 1-min denaturation step at 95 °C, a 1 min annealing step at 45 °C and a 1-min extension step at 72 °C. The first cycle was preceded by an initial step at 95 °C for 5 min. After 35 cycles, there was a final 7-min extension step at 72 °C. Negative controls without DNA template were included in parallel. PCR products were separated in 1.5% (*w/v*) agarose gel (Sigma-Aldrich) by electrophoresis for 45 min at 120 V in TBE 0.5 × (Sigma-Aldrich) and were subsequently visualized by UV illumination after ethidium bromide (50 μg/mL) staining (Sigma-Aldrich).

The PCR amplicons obtained from the amplification of the V1 region of 16S rRNA were separated by denaturing gradient gel electrophoresis (DGGE) using the Biorad DCode™ Universal Mutation Detection System (BioRad, Hercules, CA, USA) as described by Testa et al. [36]. Electrophoresis was performed in a 0.8-mm polyacrylamide gel (8% *w/v* acrylamide-bisacrylamide 37.5:1) by using two different ranges of denaturant to optimize the separation of the products. Two denaturant gradients, from 40 to 60% (100% denaturant was 7 M urea plus 40% *w/v* formamide) increasing in the direction of electrophoresis run, were used. The gels were subjected to a constant voltage of 120 V for 5 h at 60 °C, and after electrophoresis they were stained for 20 min in 1.25 × TAE containing 50 μg/mL ethidium bromide and visualized under UV illumination. DGGE gels were digitally captured by GEL DOC XR System (Bio-Rad) using the software Quantity One Analysis Version 4.6.7 (Bio-Rad) and analyzed with the pattern analysis software package, Gel Compare II Version 2.0 (Applied Maths, Kortrijk, Belgium). The calculation of similarities in the profiles of bands was based on a Pearson product-moment correlation coefficient. Dendrograms were obtained by mean of the Unweighted Pair Group Method using Arithmetic Average (UPGMA) clustering algorithm.

#### 2.4.3. DNA Sequencing and Data Processing

Representative isolate from each cluster, obtained by DGGE analysis, was amplified with primers P1V1 (5′-GCG GCG TGC CTA ATA CAT GC-3′) and P4V3 (5′-ATC TAC GCA TTT CAC CGC TAC-3′), as described by Klijn et al. [37], targeting 700 bp of the V1–V3 region of the 16S rRNA gene. After purification (QIAquick PCR purification kit, QIAGEN GmbH, Hilden), products were sent to a commercial facility for sequencing (Eurofins MWG Biotech Company, Ebersberg, Germany). Sequences were aligned with those in GeneBank utilising the Blast program [38] to determine the closest known relatives, based on the partial 16S rRNA gene homology.

#### 2.4.4. RAPD-PCR Analysis

The strains belonging to *Al. kunkei* (85), *Lp. plantarum* (61) and to *F. fructosus* (16) were biotyped using Randomly Amplified Polymorphic DNA-Polymerase Chain Reaction (RAPD-PCR) analysis. For this purpose, amplification reactions were performed in a 25 μL reaction volume containing 10 mmol/L Tris–HCl (pH 8.3), 50 mmol/L KCl, 200 μmol/L of each dATP, dGTP, dCTP and dTTP, 1.5 mmol/L MgCl_2_, 1 μmol/L primer, 80 ng DNA and 1.25 U *Taq*-DNA polymerase (Thermo Fisher Scientific). A Master cycler gradient (Eppendorf, Hamburg, Germany) was used with the following primers and amplification conditions: (a) M13: 5′-GAGGGTGGCGGTTCT-3′ [39]—the amplification was carried out for 35 cycles of 94 °C for 1 min, 40 °C for 20 s, ramp to 72 °C at 0.5 °C/s, 72 °C for 2 min; (b) D8635: 5′-GAGCGGCCAAAGGG AGCAGAC-3′ [40]—after an initial step of 94 °C for 2 min the amplification was performed for 35 cycles of 94 °C for 1 min, 42 °C for 1 min, 72 °C for 1 min and 30 s, and a final step at 72 °C for 10 min. At least three independent amplification reactions were performed for each primer. The amplicons were separated by means of electrophoresis runs on 1.5% (*w/v*) agarose gel (Sigma-Aldrich) in 0.5 × TBE buffer. Then, the gels were stained with ethidium bromide, digitalized using the GEL DOC XR System (Bio-Rad) and analyzed with the software Gel Compare II Version 2.0 (Applied Maths, Kortrijk, Belgium). A whole-image subtraction background was performed and filters were applied in order to remove gel opacity and random signal noise, which can interfere with data analysis. Bands were automatically detected according to the software wizard. Then, manual band editing was carried out to remove artefacts that were not individuated with the filtering process. Each RAPD-PCR profile was scored for the presence or absence of amplification products. Prior to compare the RAPD-PCR profiles between the different isolates, the reproducibility of the experimental conditions was estimated. For this purpose, a reproducibility index (RI) was calculated dividing the number of bands common to all replicates (x, y, z, …*i*) in the RAPD-PCR profile (n_xyz …*i*_) by the number of scored bands for all replicates. In detail the following relation was used: RI = Xn_xyz …*i*_/ (n_x_+ n_y_+ n_z_ + n …*_i_*) where X is the number of replicates, n_x_, n_y_, n_z_ and n*_i_* represent the number of bands in the all replicates. The bands that were shared in all replicates determining an RI of at least 90% were selected as reliable bands and used to make similarity matrices. Similarity matrices were generated comparing the similarity between the lanes. For this purpose, the Dice similarity coefficient with a tolerance of 10 was computed. Similarity matrices were used to generate dendrograms based on hierarchical clustering, using the UPGMA algorithm. Samples were considered to be of the same strain within a species when similarity values were ≥ 85%.

### 2.5. Biochemical Characterization

All the strains belonging to *Al. kunkei*, *Lp. plantarum* and to *F. fructosus* were assessed for their carbohydrate fermentation pattern using API 50CHL system kit and enzymatic patterns using API ZYM system kit, according to the manufacturer’s instructions (bioMérieux SA, Marcy l’Etoile, France).

## 3. Results and Discussion

### 3.1. Genetic Characterization of LAB

#### 3.1.1. Species Diversity (Species Identification)

One hundred and sixty-nine isolates based on Gram staining, a catalase test and morphological analysis were presumptively identified as LAB. The species-level taxonomic placement was performed thought a DGGE-PCR analysis.

**Figure 1:**
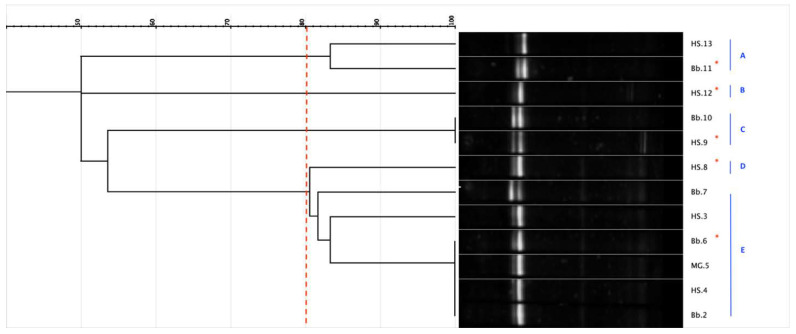
Representative image regarding the cluster analysis of Denaturing Gradient Gel Electrophoresis (DGGE) profiles of 169 strains, isolated from beebread, midgut and honey stomach. Blue capital letters indicate the cluster obtained with a threshold of 80% of similarity (red dashed line). * indicates the strains representative for each cluster subjected to sequencing of V1–V3 region of the 16S rRNA gene.

Considering a similarity level of 80% as the arbitrary threshold for the identification at species level, the isolates were grouped into clusters. According to the migration profiles, for each DGGE-gel, one strain from each cluster and all strains grouping alone were selected for subsequent genetic sequencing. In Figure 1 a representative image of the DGGE profiles cluster analysis was reported and the strains subjected to sequencing were indicated.

A total of 45 isolated were sequenced and the results (Table S1) allowed for identification at the species level. Combining these results with those obtained from the DGGE profiles cluster analysis, it was possible to identify 85 isolates as *Apilactobacillus kunkeei* (*Al. kunkeei*), 61 as *Lactiplantibacillus plantarum* (*Lp. plantarum*), 16 as *Fructobacillus fructosus* (*F. fructosus*), 6 as *Levilactobacillus brevis* (*Lv. brevis*) and 2 as *Lactobacillus delbrueckii subsp. lactis* (*Lb. lactis*). Results from sequencing and DGGE clustering analysis, guaranteeing LAB identification at the species level, highlighted a low inter-species variability in the LAB community. In fact, the substantial predominance of *Al. kunkeei* and *Lp. plantarum* was detected.

The presence of these species was in agreement with other studies on honey bee microbiota [41,42]. *Lp. plantarum*, due to its ability to cope with different stress conditions, is a versatile and widespread microorganism found in different food matrices and environments [43,44,45,46,47]. *Al. kunkeei* colonizes fructose-rich niches (e.g., flowers, fruits, and fermented foods made from fruits) and is actually classified as a fructophilic lactic acid bacterium (FLAB) [20,23]. Our results highlighted that the community of LAB also included a significant presence of isolated *F. fructosus*. This species, as also reported by other authors, dominate long-stored beebread and honey bee crop [48].

#### 3.1.2. Species Distribution

**Figure 2:**
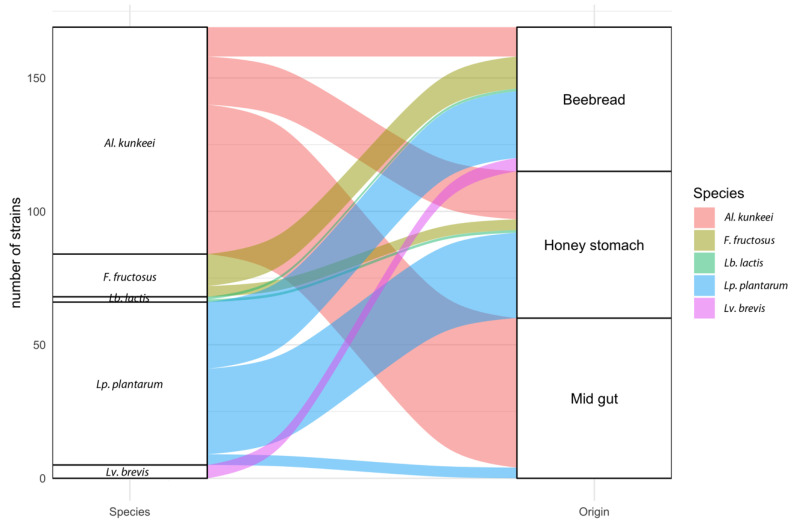
Alluvial plot showing the frequency of strains belonging to the five lactic acid bacteria species isolated from the beebread, honey stomach and midgut. The sizes of the boxes used for bacteria and boxes used for sources of isolation is proportional to the number of isolates present in the compartments (54 strains in beebread, 55 in honey stomach and 60 in midgut).

The levels of inter-species variability depend on the isolation sources: beebread, honey stomach and midgut (Figure 2). The beebread highlighted the presence of all the detected species. Specifically, *Al. kunkeei*, *Lp. plantarum* and *F. fructosus* were detected as predominant species and *Lv. brevis* and *Lb. lactis* as a minority. In honey stomach the presence of isolates belonging to four species were detected. In honey stomach *Al. kunkeei* and *Lp. plantarum* represented the predominant species, while isolates identified as *F. fructosus* as well as *Lb. lactis* were the minority. Finally, in midgut the presence of only two species was detected and *Al. kunkeei* represent the predominant specie. In fact, 56 isolates were identified as *Al. kunkeei* and only four isolates as *Lp. plantarum*.

The presence of several species in beebread is due to numerous factors including the host itself, the environment as well as the flowers [16,49,50]. In particular, the high presence of fructophilic LAB (FLAB) species could be due to the specific acacia pollen characterized by a high frucotse/glucose ratio [51,52]. In fact, *F. fructosus* and *Al. kunkeei* could be considered usual FLAB species and *Lp. plantarum* has been described as a potentially new FLAB [20]. As also reported by others authors, it is reasonable to hypothesize that FLAB species could be considered members primarily associated with foraged foods and consequently transient member of the bee-associated gut [16,48]. In addition, the selective pressure of honey bees’ gastrointestinal tract (GIT) establishes a great decrease in species variability [2,53]. In fact, as clearly highlighted by the results (Figure 2), only *Al. kunkeei* was able to persist in midgut. The high persistence of *Al. kunkeei* could be attributable to its ability to produce biofilms or to other metabolic and specific adaptability.

#### 3.1.3. Strain Diversity

**Figure 3:**
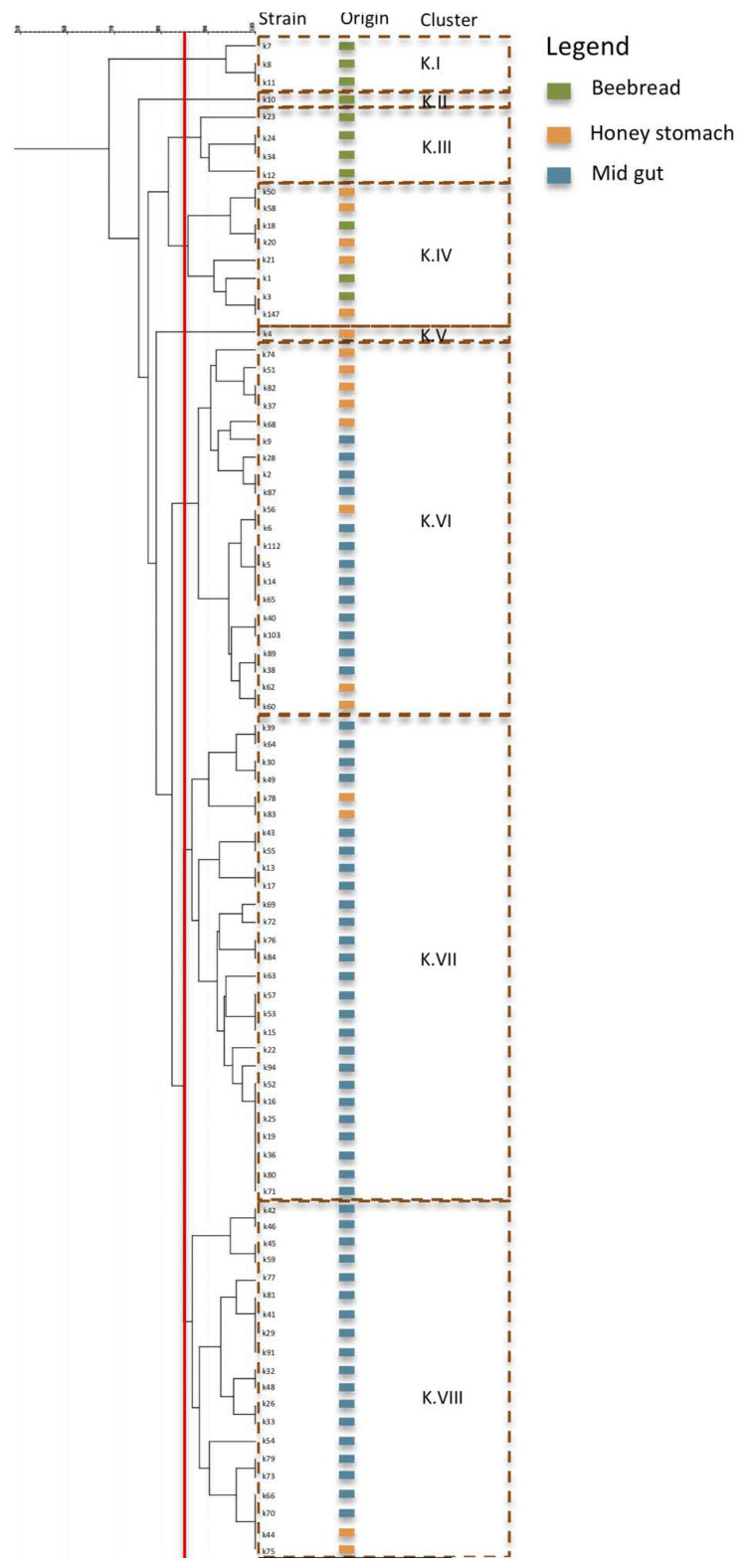
Cluster analysis based on the Randomly Amplified Polymorphic DNA-Polymerase Chain Reaction (RAPD-PCR) profiles of the 85 strains belonging to *Apilactobacillis kunkeei*. Vertical red line indicates the threshold value (85%).

Based on the previous evidence, we tried to obtain a more in-depth knowledge of LAB diversity. The target of the investigation was represented by *Lp. plantarum*, *Al. kunkeei* and *F. fructosus*, the main species isolates in different environments. For this purpose, the RAPD-PCR analysis was used to detect the distribution in relation to the isolation from different environments. The RAPD-PCR enabled strain differentiation within species, according to the PCR patterns amplified randomly with specific oligonucleotides M13 and D8635. Considering the migration profiles, a similarity level of 85% was chosen to distinguish the different biotypes for each species. In detail, 85 strains of *Al. kunkeei*, including 56 from midgut, 18 from honey stomach and 11 strains from beebread, were analyzed and, considering the profiles of bands and the cluster analysis results (Figure 3), were grouped into eight clusters (K.I–K.VIII). The results showed a relationship between the origin of the isolates and their distribution in different clusters. In particular, three biotypes, grouped in cluster K.I, K.II and K.III, respectively, were found exclusively in beebread and one biotype (cluster K.V) was detected exclusively in honey stomach. Conversely, one biotype grouped in cluster K.IV was detected in beebread and in honey stomach, and three biotypes, enclosed in cluster K.VI, K.VII and KVIII, were revealed in honey stomach and midgut. Therefore, it clear that the isolation environment not only influences the distribution of the species but also the strain selection. Our findings enriched the scientific knowledge. To date the structure of the gut microbiome of *A. mellifera* is well known as well as the dynamic change across different developmental stages [2,41,54]. So far, an increase in *Lp. kunkeei* strains according to the age of the bees and their presence in several organs of foragers was detected [41,53,55]. However, non-information on *Lp. kunkeei* has been reported. Our results highlighted for the first time a biodiversity in *Lp. kunkeei* and the relationship between biotypes and specific environments.

**Figure 4:**
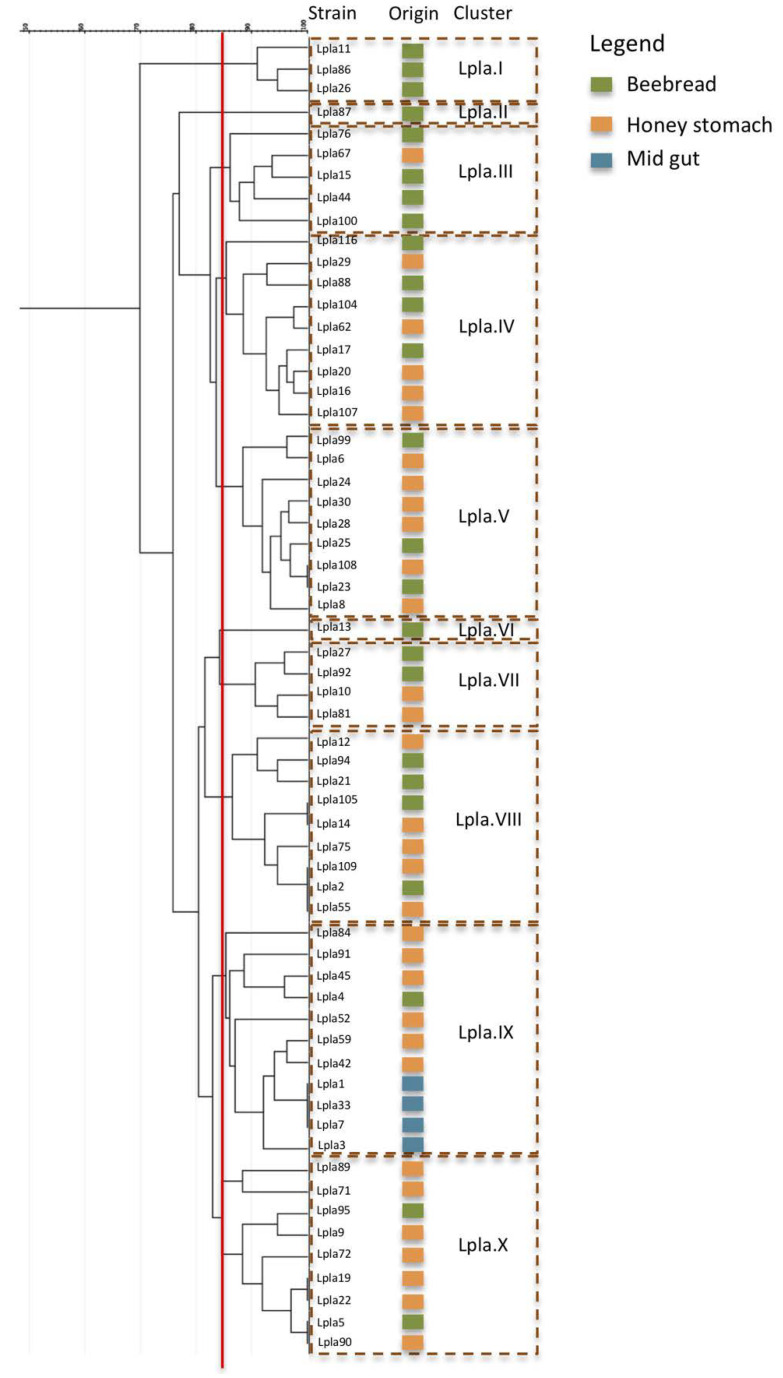
Cluster analysis based on the RAPD-PCR profiles of the 61 strains belonging to *Lactiplantibacillus plantarum.* Vertical red line indicates the threshold value (85%).

In Figure 4, the typing diversity among 61 *Lp. plantarum* strains isolated from beebread (11), honey stomach (18) and from midgut (56) was reported. In detail, 10 clusters (Lpla.I–Lpla.X) were individuated and, with respect to *Al. kunkeei*, a less significant relationship between the source of isolation and biotyping was ascertained. In fact, only three clusters (Lpla.I, Lpla.II, Lpla.VI) grouped strains came from a unique environment. The others seven clusters enclosed strains from multiple environments. In more detail, six clusters (Lpla.III–Lpla.V, Lpla.VII-VIII and Lpla.X) enclosed strains from beebread and honey stomach. Finally, cluster IX grouped strains from all the isolation environments, four from midgut, six from honey stomach and one from beebread. Based on the results, a high *Lp. plantarum* biodiversity was detected in beebread and in honey stomach, while a specialization characterized the midgut that was dominated by only one *Lp. plantarum* biotype.

**Figure 5:**
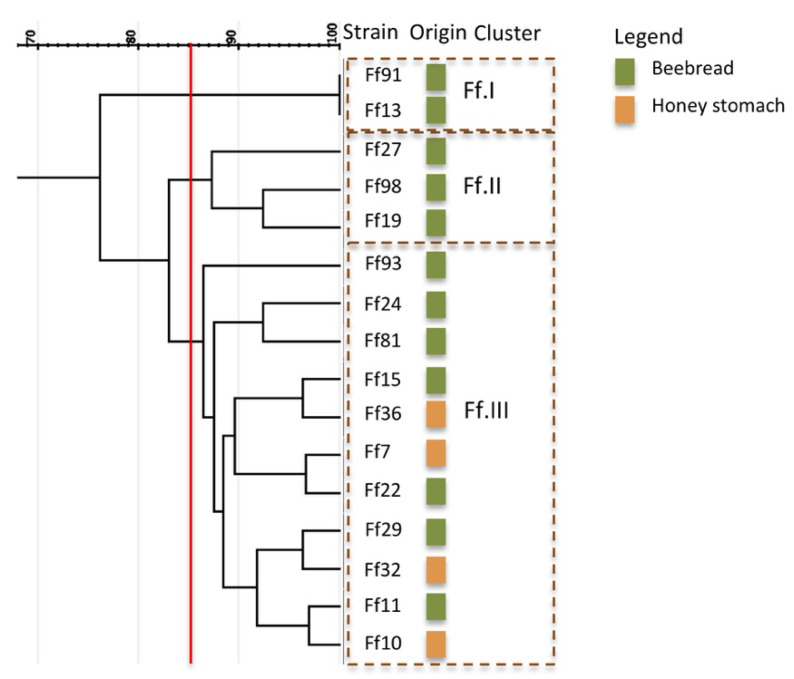
Cluster analysis based on the RAPD-PCR profiles of the 16 strains belonging to *Fructobacillus fructosus.* Vertical red line indicates the threshold value (85%).

*F. fructosus* strains, isolated from beebread and honey stomach, as reported in Figure 5, were grouped into three clusters (Ff.I, Ff.II, Ff.III). Cluster Ff.III showed strains from both environments, while clusters Ff.I and Ff.II grouped only strains isolated from beebread. Even if distinct clusters were detected, it should be considered that the number of samples (isolates) for this species is much lower than for the previous species (*Al. kunkeei*, *Lp. plantarum*).

The results showed that several biotypes from each species populated the beebread highlighting a great intra-species biodiversity. Conversely, in the midgut of only *Al. kunkeei* was a fair amount of biodiversity observed.

### 3.2. Biochemical Characterization

**Figure 6:**
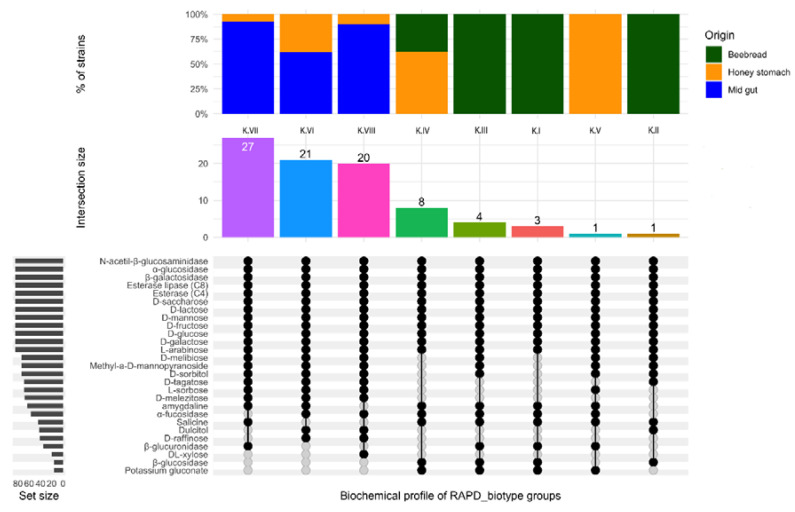
Upset plot regarding the biochemical profile of the 8 RAPD_biotype groups belonging to *Apilactobacillis kunkeei*. Vertical barplot indicates the number of overlapping strains (intersection size) for each intersection represented by the connected dots. The intersection matrix is sorted in descending order. The grey horizontal barplots, reported as “Set size”, indicate the number of strains showing enzymatic activity.

**Figure 7:**
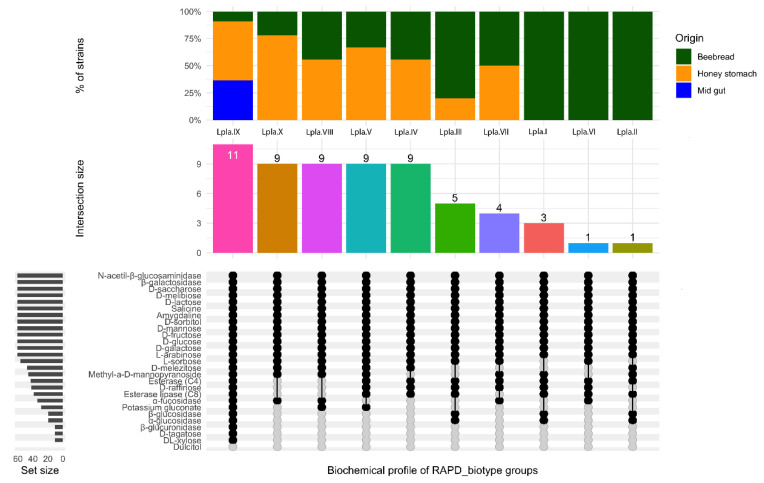
Upset plot regarding the biochemical profile of the 10 RAPD_biotype groups belonging to *Lactiplantibacillus plantarum*. Vertical barplot indicates the number of overlapping strains (intersection size) for each intersection represented by the connected dots. The intersection matrix is sorted in descending order. The grey horizontal barplots, reported as “Set size”, indicate the number of strains showing enzymatic activity.

The assessment of biochemical profiles attributable to different strains allowed, for each species, the individuation of specific features as well as important differences related to the RAPD_biotypes. In fact, the results highlighted that each RAPD_biotype was characterized by a specific biochemical profile. Moreover, interesting metabolic activities were found both in *Al. kunkeei* and in *Lp. plantarum* (Figure 6 and Figure 7, respectively). The enzyme profiles showed that all the strains belonging to *Al. kunkeei* (Figure 6) were able to utilize not only the three most common nectar sugars (glucose, fructose, and sucrose) but also arabinose, galactose, mannose and lactose, described as toxic to honey bees [56,57]. Already other authors had reported that the honey bee gut microbiome may facilitate the metabolism of specific toxic sugars [24,58,59].

The results also evidenced that all the *Al. kunkeei* strains possessed several enzymes such as esterase, esterase lipase and alpha-glycosidase. This last enzyme converts maltose to glucose and is also involved, together with alpha-amylase, in the degradation of starch granules. In addition to the above species-specific biochemical traits, a strain-specific characteristic was found. In particular, based on their biochemical profiles, six groups were individuated and two strains were unique. The main strains (80%), all isolates from the midgut and from honey stomach, were grouped into three cluster and the RAPD_biotypes K.VII, K.VI and K.VIII were collected, respectively. All the strains from these RAPD_biotypes metabolized the monosaccharides sorbose and tagatose and the trisaccharide melezitose. The ability to use this last oligosaccharide is of particular interest. In fact, melezitose, composed by glucose and turanose and produced by aphids, is known to be poorly assimilated by honey bees and leads to increased losses of winter colonies fed on honeydew honey. Moreover, when present in high amounts, it plays a key role in the occurrence of the honeydew flow disease in bee colonies. Biochemical activities expressed by the RAPD_biotype K.VIII was also enriched by the ability to metabolize xylose and raffinose, two sugars that the scientific literature described as toxic to bees due to the absence of appropriate enzymes for their digestion [58]. The RAPD_biotype K.VII differed from the RAPD_biotypes K.VI and K.VIII in terms of its inability to utilize the dulcitol and raffinose as well as for the presence of the β-glucuronidase enzyme. The RAPD_biotypes K.I, K.II, K.III and K.IV (Figure 6), all isolates from beebread or from honey stomach, are distinguished by β-glucosidase activities. The β-glucosidase hydrolyzes the glycosylated aromatic precursors, which releases odorous compounds including monoterpenes and increases the bioavailability of antioxidative plant metabolites in honey [24,60,61]. In addition, β-glucosidase is important because, in combination with other enzymes, including cellulase and hemicellulase, it contributes to hydrolyze cellulose [59]. RAPD_biotypes K.I and K.IV differed with respect to K.II and K.III in terms of their inability to utilize sorbitol and methyl-α-D-mannopyranoside as well as melibiose, also considered toxic to bees [62]. Moreover, the RAPD_biotype K.II was characterized for the ability to metabolize dulcitol and tagatose and for the absence of β-glucuronidase and α-fucosidase enzymes. A specific biochemical profile also characterized the RAPD_biotype K.V, specific for honey stomach, which showed the ability to metabolize certain sugars such as sorbose, melibiose and possessed β-glucuronidase and α-fucosidase enzymes. The results highlighted that different *Al. kunkeei* biotypes were associated with different organs of *Apis millifera* and with beebread. Moreover, each biotype with its biochemical profiles cold contribute to the physiology of the specific organ (e.g., to improve the absorption of certain sugar) or to the characteristics of beebread. A relationship between RAPD_biotypes and biochemical features was also detected for *Lp. plantarum* strains (Figure 7).

**Figure 8:**
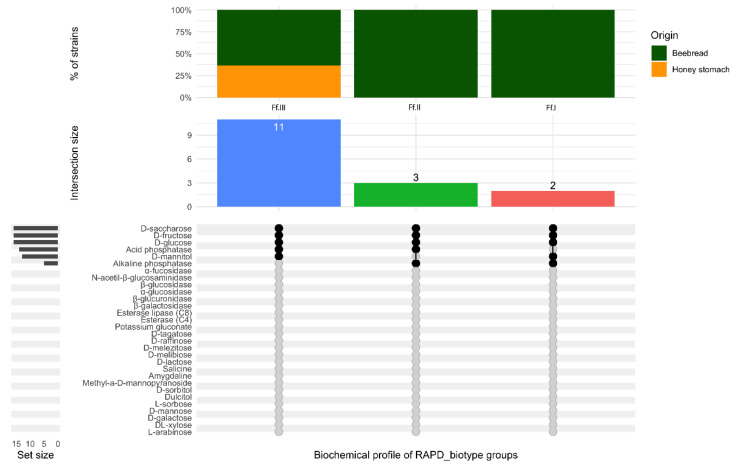
Upset plot regarding the biochemical profile of the 3 RAPD_biotype groups belonging to *Fructobacillus fructosus*. Vertical barplot indicates the number of overlapping strains (intersection size) for each intersection represented by the connected dots. The intersection matrix is sorted in descending order. The grey horizontal barplots, reported as “Set size”, indicate the number of strains showing enzymatic activity.

Based on the strain-dependent biochemical features ten groups were identified. Three cluster, collected the three RAPD_biotypes (Lpla.I, Lpla.II and Lpla.VI) detected exclusively in beebread, exhibited several enzymatic activities including esterase, esterase lipase, α-glucosidase and β-glucosidase. RAPD_biotypes Lpla.I and Lpla.VI were also characterized by the ability to utilize raffinose; while the RAPD_biotype II metabolized melezitose. Six different biochemical clusters grouped the six *Lp. plantarum* RAPD_biotypes found in beebread and in honey stomach. Final, regarding *Lp. plantarum* strains, a specific biochemical cluster collected the RAPD_biotype Lpla.IX. The results showed that this biotype, detected not only in beebread and honey stomach but also in midgut, was characterized by the most complex biochemical profile. This last profile was able to metabolize the most common nectar sugar as well as the sugar described as toxic or poorly assailable to bees [58]. Moreover, the RAPD_biotype Lpla.IX showed α-glucosidase, β-glucosidase activities and possessed esterase and esterase lipase enzymes. It is reasonable to assume that this metabolic complexity makes the biotype Lpla.IX particularly versatile and adaptable to the different assayed environments. This biotype is particularly interesting as it plays a simultaneous role in the breakdown of complex polysaccharides and metabolizes toxic sugars; the role of these *Lp. plantarum* strains in improving dietary tolerance as well maintaining the health of their hosts might be notable [63,64,65]. Finally, also the RAPD_biotypes of *F. fructosus*, as reported in Figure 8, were characterized by a specific biochemical profile highlighting a discrete biodiversity.

## 4. Conclusions

The present research strongly enriches the knowledge on *A. mellifera* microbiota. So far, several authors have described the inter-species diversity in *A. mellifera* subspecies [20,48,66,67,68,69]. Our results showed a low biodiversity in species confirming the predominance of *Al. kunkeei* and *Lp. plantarum* in the social stomach and in midgut. *F. fructosus*, along with *Al. kunkeei* and *Lp. plantarum*, were mainly associated with beebread. For all the three species a reasonable intra-species biodiversity was found and a relationship between biotypes and compartments has been detected. An exhaustive study on genomic diversity of honey bee gut microbiota has been reported by Ellegaard and Engel [70] evidencing that the bacterial population from honey bee guts are not only organized into discrete genetic clusters, so-called “sequence-discrete populations” (SDPs), but each SDP of the honey bee gut microbiota harbor very high levels of strain diversity. However, as also evidenced by other authors [71,72,73], to identify the causes and consequences of intra-species diversity, experimental and phenotypic data must be linked to each biotype profile. Moreover, with our knowledge, only few studies [74,75], if any, link the biotype’s diversity to specific ecological niches in honey bee foragers. On this basis, the main important scientific result obtained in our study was the finding that the specific biotypes, through their metabolic profile, contribute to the correct physiology of the associated honey bee organs or to the definition of beebread features. In fact, regardless of species, the biotypes associated with the social stomach and midgut were able to metabolize several sugars considered toxic to bees. In addition, the biotypes detected in beebread could contribute to the release of odorous compounds or to the increase in antioxidative metabolites.

The inter- and intra-species diversity of LAB-specific as well as functional diversity could present a new horizon to consider the honey bee microbiome. Therefore, the results obtained in this study could be considered as important knowledge for the development of biotechnological approaches able to improve the health of honey bees or to enhance the quality features of their products.

## Supplementary Materials

The following are available online at https://www.mdpi.com/2076-2607/8/10/1578/s1, Table S1. Lactic Acid Bacteria identification at specie level, based on blast camparison in GenBank, of 45 strains selected on basis DGGE cluster analysis.
