# Supplementary material for: Inter- and Intra-Species Diversity of Lactic Acid Bacteria in Apis mellifera ligustica Colonies
Source: Microorganisms. 2020 Oct 14;8(10):1578. doi: 10.3390/microorganisms8101578 (PMC7602248; doi:10.3390/microorganisms8101578)
Supplement: Supplementary file 1 [file microorganisms-08-01578-s001.pdf]

**Table S1.** Lactic Acid Bacteria identification at specie level, based on blast camparison in GenBank, of 45 strains selected on basis DGGE cluster analysis

| Strains | Origin | Closest relative | % Identity | Sequences | Source* |
|---|---|---|---|---|---|
| Lpla11 | bee bread | *Lp. plantarum* | 98.64 | AAAAGTCGAACGAACTCTGGTATTGATTGGTGCTTGCATCATGATTTAC<br>ATTTGAGTGAGTGGCGAACTGGTGAGTAACACGTGGGAAACCTGCCCA<br>GAAGCGGGGGATAACACCTGGAAACAGATGCTAATACCGCATAACAA<br>CTTGGACCGCATGGTCCGAGCTTGAAAGATGGCTTCGGCTATCACTTTT<br>GGATGGTCCCGCGGCGTATTAGCTAGATGGGGGGGTAACGGCTCACCA<br>TGGCAATGATACGTACCCGACCTGAGAGGGTAATCGGCCACATTGGGA<br>CTGAAACACGGCCCAAACTCCTACGGGAGGCAGCAGTAGGGAATCTTC<br>CACAATGGACGAAAGTCTGATGGAGCAACGCCGCGTGAGTGAAAAAG<br>GGTTTCGGCTCGTAAAACTCTGTTGTTAAAAAAAAACATATCTGAAAG<br>TAACTGTTCAGGTATTGACGGTATTTAACCAGAAAGCCACGGCTAACT<br>ACGTGCCAGCAGCCGCGGTAATACGTAGGTGGCAAGCGTTGTCCGGAT<br>TTATTGGGCGTAAAGCGAGCGCAGGCGGTTTTTTAAGTCTGATGTGAA<br>AGCCTTCGGCTCAACCGAAAAGTGCATCGGAAACTGGGAAACTTGAGT<br>GCAAAAAAGGACAGTGGAACTCCATGTGTAGCGGTGAAATTGC | **MT184183.1** |
| Lpla87 | bee bread | *Lp. plantarum* | 97.71 | AAGTCGAACGAACTCTGGTATTGATTGGGGCTTGCATCATGATTTACAT<br>TTGAGTGAGTGGCGAACTGGTGAGTAACACGTGGGAAACCTGCCCAAA<br>AGCGGGGGATAACACCTGGAAACAGATGCTAATACCGCATAACAACTT<br>GGACCGCATGGTCCGAGCTTGAAAGATGGCTTCGGCTATCACTTTTGG<br>ATGGTCCCGCGGCGTATTACCTAAATGGGGGGGTAACGGCTCACCATG<br>GCAATGATACGTACCCGACCTGAAAGGGTAATCGGCCACATTGGGACT<br>GAAACACGGCCCAAACTCCTACGGGAGGCAGCAGTAGGGAATCTTCCA<br>CAATGGACAAAAGTCTGATGGAGCAACGCCGCGTGAGTGAAAAAGGG<br>TTTCGGCTCGTAAAACTCTGTTGTAAAAAAAAACATATCTGAGAGTAA<br>CTGT | **AY341564.1** |

| | | | | | |
|---|---|---|---|---|---|
| Lpla88 | bee bread | *Lp. plantarum* | 98.33 | TGATTGGTGCTTGCATCATGATTTACATTTGAGTGAGTGGCGAACTGGT<br>GAGTAACACGTGGGAAACCTGCCCAAAAGCGGGGGATAACACCTGGA<br>AACAGATGCTAATACCGCATAACAACTTGGACCGCATGGTCCGAGCTT<br>GAAAGATGGCTTCGGCTATCACTTTTGGATGGTCCCGCGGCGTATTAGC<br>TAAATGGGGGGGTAACGGCTCACCATGGCAATGATACGTACCCGACCT<br>GAGAGGGTAATCGGCCACATTGGGACTGAAACACGGCCCAAACTCCTA<br>CGGGAGGCAGCAGTAGGGAATCTTCCACAATGGACGAAAGTCTGATG<br>GAGCAACGCCGCGTGAGTGAAAAAGGGTTTCGGCTCGTAAAACTCTGT<br>TGTTAAAGAAAAACATATCTGAAAGTAACTGTTCAG | **MG754599.1** |
| Lpla100 | bee bread | *Lp. plantarum* | 99.12 | TGATTGGTGCTTGCATCATGATTTACATTTGAGTGAGTGGCGAACTGGT<br>GAGTAACACGTGGGAAACCTGCCCAGAAGCGGGGGATAACACCTGGA<br>AACAGATGCTAATACCGCATAACAACTTGGACCGCATGGTCCGAGCTT<br>GAAAGATGGCTTCGGCTATCACTTTTGGATGGTCCCGCGGCGTATTAGC<br>TAGATGGTGGGGTAACGGCTCACCATGGCAATGATACGTACCCGACCT<br>GAGAGGGTAATCGGCCACATTGGGACTGAAACACGGCCCAAACTCCTA<br>CGGGAGGCAGCAGTAGGGAATCTTCCACAATGGACGAAAGTCTGATG<br>GAGCAACGCCGCGTGAGTGAAAAAGGGTTTCGGCTCGTAAAACTCTGT<br>TGTTAAAGAAAAACATATCTGAAAGTAACTGTTCAGGTATTGACGGTA<br>TTTAACCAGAAAGCCACGGCTAATT | **MT184183.1** |
| Lpla25 | bee bread | *Lp. plantarum* | 98.49 | TTAAAGTCGAACGAACTCTGGTATTGATTGGGGCTTGCATCATGATTTA<br>CATTTGAGTGAGTGGCGAACTGGTGAGTAACACGTGGGAAACCTGCCC<br>AAAAGCGGGGGATAACACCTGGAAACAGATGCTAATACCGCATAACA<br>ACTTGGACCGCATGGTCCGAGCTTGAAAGATGGCTTCGGCTATCACTTT<br>TGGATGGTCCCGCGGCGTATTAGCTAGATGGTGGGGTAACGGCTCACC<br>ATGGCAATGATACGTACCCGACCTGAAAGGGTAATCGGCCACATTGGG<br>ACTGAAACACGGCCCAAACTCCTACGGGAGGCAGCAGTAGGGAATCTT<br>CCACAATGGACAAAAGTCTGATGGAGCAACGCCGCGTGAGTGAAAAA<br>GGGTTTCGGCTCGTAAA | **MH665755.1** |
| Lpla26 | bee bread | *Lp. plantarum* | 97.06 | GCATGGTCCGAGCTTGAAAGATGGCTTCGGCTATCACTTTTGGATGGCC<br>CCGCGGCGTATTAGCTAAATGGGGGGGTAACGGCTCACCATGGCAATG<br>ATACGTACCCGACCTGAAAGGGTAATCGGCCACATTGGGACTGAAACA<br>CGGCCCAAACTCCTACGGGAGGCAGCAGTAGGGAATCTTCCACAATGG<br>ACAAAAGTCTG | **MT299623.1** |

| | | | | | |
|---|---|---|---|---|---|
| Lpla27 | bee bread | *Lp. plantarum* | 98.72 | CGAACGAACTCTGGTATTGATTGGTGCTTGCATCATGATTTACATTTGA<br>GTGAGTGGCGAACTGGTGAGTAACACGTGGGAAACCTGCCCAGAAGC<br>GGGGGATAACACCTGGAAACAGATGCTAATACCGCATAACAACTTGGA<br>CCGCATGGTCCGAGCTTGAAAGATGGCTTCGGCTATCACTTTTGGATGG<br>TCCCGCGGCGTATTAGCTAGATGGGGGGGTAACGGCTCACCATGGCAA<br>TGATACGTACCCGACCTGAAAGGGTAATCGGCCACATTGGGACTGAAA<br>CACGGCCCAAACTCCTACGGGAGGCAGCAGTAGGGAATCTTCCACAAT<br>GGACAAAAGTCTGATGGAGCAACGCCGCGTGAGTGAAAAAGGGTTTC<br>GGCTCGTAAAACTCTGTTGTTAAAGAAAAACATATCTGAAAGTAACTG<br>TTCAGGTATTGACGGTATTTAACCAGAAAGCCACGGCTAACTACGTGC<br>CAGCAGCCGCGGTAATACGTAGGTGGCAAGCGTTGTCCGGATTTATTG<br>GGCGTAAAGCGAGCGCAGGC | **MH665755.1** |
| Lpla92 | bee bread | *Lp. plantarum* | 98.99 | ATTGGATTGGTGCTTGCATCATGATTTACATTTGAGTGAGTGGCGAACT<br>GGTGAGTAACACGTGGGAAACCTGCCCAGAAGCGGGGGATAACACCT<br>GGAAACAGATGCTAATACCGCATAACAACTTGGACCGCATGGTCCGAG<br>CTTGAAAGATGGCTTCGGCTATCACTTTTGGATGGTCCCGCGGCGTATT<br>AGCTAGATGGTGGGGTAACGGCTCACCATGGCAATGATACGTACCCGA<br>CCTGAGAGGGTAATCGGCCACATTGGGACTGAAACACGGCCCAAACTC<br>CTACGGGAGGCAGCAGTAGGGAATCTTCCACAATGGACGAAAGTCTGA<br>TGGAGCAACGCCGCGTGAGTGAAAAAGGGTTTCGGCTCGTAAAACTCT<br>GTTGTTAAAGAAAAACATATCTGAGAGTAACTGTTCAGGTATTGACGG<br>TATTTAACCAGAAAGCCACGGCTAACTACGTGCCAGCAGCCGCGGTAA<br>TACGTAGGTGGCAAGCGTTGTCCGGATTTATTGGGCGTAAAGCGAGCG<br>CAGGCGGTTTTTTAAGTCTGATGTGAAAGCCTTCGGCTCAACCGAAAA<br>AGTGCATCGGACACT | **MT510329.1** |
| Lpla94 | bee bread | *Lp. plantarum* | 98.99 | GGTGCTTGCATCATGATTTACATTTGAGTGAGTGGCGAACTGGTGAGT<br>AACACGTGGGAAACCTGCCCAGAAGCGGGGGATAACACCTGGAAACA<br>GATGCTAATACCGCATAACAACTTGGACCGCATGGTCCGAGCTTGAAA<br>GATGGCTTCGGCTATCACTTTTGGATGGTCCCGCGGCGTATTAGCTAAA<br>TGGGGGGGTAACGGCTCACCATGGCAATGATACGTACCCGACCTGAGA<br>GGGTAATCGGCCACATTGGGACTGAAACACGGCCCAAACTCCTACGGG<br>AGGCAGCAGTAGGGAATCTTCCACAATGGACGAAAGTCTGATGGAGC<br>AACGCCGCGTGAGTGAAAAAGGGTTTCGGCTC | **MT510329.1** |

| | | | | | |
|---|---|---|---|---|---|
| Lpla95 | bee bread | *Lp. plantarum* | 99.06 | AAAGTCGAACGAACTCTGGTATTGATTGGTGCTTGCATCATGATTTACA<br>TTTGAGTGAGTGGCGAACTGGTGAGTAACACGTGGGAAACCTGCCCAG<br>AAGCGGGGGATAACACCTGGAAACAGATGCTAATACCGCATAACAAC<br>TTGGACCGCATGGTCCAAGTTTGAAAGATGGCTTCGGCTATCACTTTTG<br>GATGGTCCCGCGGCGTATTAGCTAGATGGTGGGGTAACGGCTCACCAT<br>GGCAATGATACGTACCCGACCTGAGAGGGTAATCGGCCACATTGGGAC<br>TGAAACACGGCCCAAACTCCTACGGGAGGCAGCAGTAGGGAATCTTCC<br>ACAATGGACGAAAGTCTGATGGAGCAACGCCGCGTGAGTGAAAAAGG<br>GTTTCGGCTCGTAAAACTCTGTTGTTAAAGAAAAACATATCTGAAAGT<br>AACTGTTCAGGTATTGACGGTATTTAACCAGAAAGCCACGGCTAACTA<br>CGTGCCAGCAGCCGCGGTAATACGTAGGTGGCAAGCGTTGTCCGGATT<br>TATTGGGCGTAAAGCGAGCGCAGGCGGTTTTTTAAGTCTGATGTGAAA<br>GCCTTCGGCTCAACCGAAAAAGTGCATCGGAAACTGGGAAACTTGAGT<br>GCAGAAAAGGACAGTGGAT | **MT184183.1** |
| k34 | bee bread | *Al. kunkeei* | 98.36 | CTCCTCCCCAAATTGATTTTATGGCTTGCATAAATGATTTTTGGATTCG<br>GAGCGAGTGGCGAACTGGTGAGTAACACGTGGGTAACCTGCCCCGAA<br>GCGGGGGATAACATTTGGAAACAAGTGCTAATACCGCATAATTAGTTG<br>GAACCGCATGGTTCCAACTTGAAAGATGGCTCTGCTATCACTTTGGGAT<br>GGACCCGCGCCGTATTAGTTAGTTGGTGAGATAAAAGCCCACCAAGAC<br>GATGATACGTAGCCGACCTGAAAGGGTAATCGGCCACATTGGGACTGA<br>AACACGGCCCAAACTCCTACGGGAGGCAGCAGTAGGGAATCTTCCACA<br>ATGGACGAAAGTCTGATGGACCAACGCCGCGTGAGTGATGAAGGTTTT<br>CGGATCGTAAAACTCTGTTGTTAAAAAAAAACAAGTGTTAAAGTAACT<br>GTTAACACTTTGACGGTATTTAACCAAAAAGCCACGGCTAACTACGTG<br>CCAGCAGCCGCGGTAATACGTAGGTGGCAAGCGTTGTCCGGATTTATT<br>GGGCGTAAAGCGAGCGCAGGCGGTTTTGTAAGTCTGCTGTGAAAGCCC<br>TCAGCTCAACTGAGGAAGTGCAGTGGATACTACAA | **KY027183.1** |
| k18 | bee bread | *Al. kunkeei* | 95.18 | AGTCGAACGAGCTCTCCCAAATTGATTTTATGCTTGCATAAATGATTTT<br>TGGATTCGGAGCGAGTGGCGAACTGGTGAGTAACACGTGGGTAACCTG<br>CCCCGAAGCGGGGGATAACATTTGGAAACAAGTGCTAATACCGCATAA<br>TTAGTTGGAACCGCATGGTTCCAACTTGAAAGATGGCTCTGCTATCACT<br>TTGGGATGGACCCGCGCCGTATTAGTTAGTTGGTGAGATAAAAGCCCA<br>CCAAGACAATGATACGTAGCCGACCTGAAAGGGTAATCGGCCACATTG<br>GGACTGAAACACGGCCCAAACTCCTACGGGAGGCAGCAGTAGGGAAT<br>CTTCCACAATGGACGAAAGTCTGATGGACCAACGCCGC | **KY027183.1** |

| | | | | | |
|---|---|---|---|---|---|
| k7 | bee bread | *Al. kunkeei* | 99.15 | TCCCAAATTGATTTTATGGCTTGCATAAATGATTTTTGGATTCGGAGCG<br>AGTGGCGAACTGGTGAGTAACACGTGGGTAACCTGCCCCGAAGCGGG<br>GGATAACATTTGGAAACAAGTGCTAATACCGCATAATTAGTTGGAACC<br>GCATGGTTCCAACTTGAAAGATGGCTCTGCTATCACTTTGGGATGGACC<br>CGCGCCGTATTAGTTAGTTGGTGAGATAAAAGCCCACCAAGACGATGA<br>TACGTAGCCGACCTGAGAGGGTAATCGGCCACATTGGGACTGAAACAC<br>GGCCCAAACTCCTACGGGAGGCAGCAGTAGGGAATCTTCCACAATGGA<br>CGAAAGTCTGATGGAGCAACGCCGCGTGAGTGATGAAGGTTTTCGGAT<br>CGTAAAACTCTGTTGTTAAAGAAAAACAAGTGTTAAAGTAACTGTTAA<br>CACTTTGACGGTATTTAACCAAAAAGCCACGGCTAACTACGTGCCAGC<br>AGCCGCGGTAATACGTAGGTGGCAAGCGTTGTCCGGATTTATTGGGCG<br>TAAAGCGAGCGCAGGCGGTTTTGTAAGTCTGCTGTGAAAGCCCTCAGC<br>TCAACTGAGGAAGTGCAGTGGAAACTACAAAACTTGAGTACAAAAGA<br>GGACAGTGGAACTCCATGTGTAGCGGTGAATG | **KY027183.1** |
| Ff91 | bee bread | *F. fructosus* | 98.92 | AAAGGGCTTGCACTTTCCAAGTAAGTGGCGAACGGGTGAGTAACACGT<br>GAATAACCTACCTCAAAGTCTGGGATAACCATTGGAAACAGTGACTAA<br>TACCGGATAAAACCCAAGTGCACATGCACTAAGGTTAAAAGCTGCGTT<br>TGCAGCGCTTTAAAATGGATTCGCGGTGCATTAGTTAGTTGGTGAGGT<br>AAAGGCTCACCAAGACGATGATGCATAGCCGAGTTGAGAGACTGACC<br>GGCCACATTGGGACTGAAACACGGCCCAAACTCCTACGGGAGGCTGCA<br>GTAGGGAATCTTCCACAATGGGCGCAAGCCTGATGGAGCAACGCCGCG<br>TGTGTGATGAAGGCTTTCGGGTCGTAAAGCACTGTTGTATGGGAAAAA<br>CGGGTTTAAAAGGAAATGCTTAAACAGTGACGGTACCATACCAAAAAG<br>GGACGGCTAAATACGTGCCAGCAGCCGCGGTAATACGTATGTCCCGAG<br>CGTTATCCGGATTTATTGGGCGTAAAGCGAGCGCAGACGGTTGCTTAA<br>GTCTGAAGTGAAAGCCCACAGCTCAACTGT | **MH796219.1** |

| | | | | | |
|---|---|---|---|---|---|
| Ff93 | bee bread | *F. fructosus* | 98.39 | GGCTTGCACTTTCCAAGTAAGTGGCGAACGGGTGAGTAACACGTGAAT<br>AACCTACCTCAAAGTCTGGGATAACCATTGGAAACAGTGACTAATACC<br>GGATAAAACCCAAGTGCACATGCACTAAGGTTAAAAGCTGCGTTTGCA<br>GCGCTTTAAAATGGATTCCCGGTGCATTATTTAGTTGGTGAGGTAAAG<br>GCTCACCAAGACGATGATGCATAGCCGAGTTGAGAGACTGACCGGCCA<br>CATTGGGACTGAAACACGGCCCAAACTCCTACGGGAGGCTGCAGTAGG<br>GAATCTTCCACAATGGGCGCAAGCCTGATGGAGCAACGCCGCGTGTGT<br>GATGAAGGCTTTCGGGTCGTAAAGCACTGTTGTATGGAAAAAACGGGT<br>TTAAAAGGAAATGCTTAAACAGTGACGGTACCATACCAAAAAGGGAC<br>GGCTAAATACGTGCCAGCAGCCGCGGTAATACGTATGTCCCGAGCGTT<br>ATCCGGATTTATTGGGCGTAAAGCGAGCGCAGACGGTTGCTTAAGTCT<br>GAAGTGAAAGCCCACAGCTCAACTGTGGAATGGCTTTGGAAACTGGGC<br>AACTTGAGTACAGTAGAGGTAAGTGGGAACTCCATGTGTAGCGGTGAT<br>TGG | **MH796219.1** |
| Ff29 | bee bread | *F. fructosus* | 98.09 | AGGGCTTGCACTTTCCAAGTAAGTGGCGAACGGGTGAGTAACACGTGA<br>ATAACCTACCTCAAAGTCTGGGATAACCATTGGAAACAGTGACTAATA<br>CCGGATAAAACCCAAGTGCACATGCACTAAGGTTAAAAGCTGCGTTTG<br>CAGCGCTTTAAAATGGATTCCCGGTGCATTATTTAGTTGGTGAGGTAAA<br>GGCTCACCAAGACGATGATGCATAGCCGAGTTGAGAGACTGACCGGCC<br>ACATTGGGACTGAAACACGGCCCAAACTCCTACGGGAGGCTGCAGTAG<br>GGAATCTTCCACAATGGGCGCAAGCCTGATGGAGCAACGCCGCGTGTG<br>TGATGAAGGCTTTCGGGTCGTAAAGCACTGTTGTATGGGAAAAACGGG<br>TTTAAAAGGAAATGCTTAAACAGTGACGGTACCATACCAAAAAGGGAC<br>GGCTAAATACGTGCCAGCAGCCGCGGTAATACGTATGTCCCGAGCGTT<br>ATCCGGATTTATTGGGCGTAAAGCGAGCGCAGACGGTTGCTTAAGTCT<br>GAAGTGAAAGCCCACAGCTTAACTGTGGAATGGCTTTGGAAACTGGGC<br>AACTGTGAGTACAGTAGAGGTAAGTGGAACTCCATGTGTAGCGGTGAA<br>TGTGGC | **MH796219.1** |
| Ff98 | bee bread | *F. fructosus* | 100 | AACAGCGGAAAGTGCTTGCACTTTCCAAGTAAGTGGCGAACGGGTGAG<br>TAACACGTGAATAACCTACCTCAAAGTCTGGGATAACCATTGGAAACA<br>GTGA | **MK986693.1** |
| 101 | bee bread | *Lv. brevis* | 100 | AAAGTCGAACGAGCTTCCGTTGAATGACGTGCTTGCACTGATTTCAAC<br>AATGAAGCGAGTGGCGAA | **MT640328.1** |
| 102 | bee bread | *Lb. lactis* | 95.77 | GGTGGGTACTTGTACCGACTGGATGACACGAACGGGTGAGTAACGCGT<br>GGGGAATCTGCCTTTGAGCGGGGGACAACATTTGGAAACAATGCTAAT<br>ACCGCATAAAAACTTTAAACACAAGTTTTACTTTGAAAAATGCTT | **KT124588.1** |

| | | | | | |
|---|---|---|---|---|---|
| 105 | bee bread | *Lv. brevis* | 95.56 | AGGAAGTCGAACGAGCTTCCGTTGAATGACGTGCTTGCACTGATTTCA<br>ACAATGAAGCGAGTGGCGAACTGGTGATTAACACGGGGGAAATCTGC<br>CCAAAACCAGGGAATAACACTTGGAAACAGGGGCTAATACCGT | **MT640328.1** |
| 107 | bee bread | *Lv. brevis* | 97.58 | AAGTCGAACGAGCTTCCGTTGAATGACGTGCTTGCACTGATTTCAACA<br>ATGAAGCGAGTGGCGAACTGGTGATTAACACGTGGGAAATCTGCCCAA<br>AAGCAGGGAATAACACTTGGAAACAGGT | **MT640328.1** |
| k41 | mid gut | *Al. kunkeei* | 100 | AGTCGAACGAGCTCTCCCAAATTGATTTTATGCTTGCATAAATGATTTT<br>TGGATTCGGAGCGAGTGGCGAACTGGTGAGTAACACGTGGGTAACCTG<br>CCCCGAAACGGGGGATAACATTTGGAAACAAGTGCTAATACCGCATAA<br>TTAGTTGGAACCGCA | **MT381736.1** |
| k45 | mid gut | *Al. kunkeei* | 99.72 | CCAAATTGATTTTATGCTTGCATAAATGATTTTTGGATTCGGAGCGAGT<br>GGCGAACTGGTGAGTAACACGTGGGTAACCTGCCCCGAAGCGGGGGA<br>TAACATTTGGAAACAAGTGCTAATACCGCATAATTAGTTGGAACCGCA<br>TGGTTCCAACTTGAAAGATGGCTCTGCTATCACTTTGGGATGGACCCGC<br>GCCGTATTAGTTAGTTGGTGAGATAAAAGCCCACCAAGACGATGATAC<br>GTAGCCGACCTGAGAGGGTAATCGGCCACATTGGGACTGAAACACGGC<br>CCAAACTCCTACGGGAGGCAGCAGTAGGGAATCTTCCACAATGGACGA<br>AAGTCTGATGGAGCAACG | **KY027183.1** |
| k52 | mid gut | *Al. kunkeei* | 99.39 | TCTCCCAAATTGATTTTATGCTTGCATAAATGATTTTTGGATTCGGAGC<br>GAGTGGCGAACTGGTGAGTAACACGTGGGTAACCTGCCCCGAAGCGG<br>GGGATAACATTTGGAAACAAGTGCTAATACCGCATAATTAGTTGGAAC<br>CGCATGGTTCCAACTTGAAAGATGGCTCTGCTATCACTTTGGGATGGAC<br>CCGCGCCGTATTAGTTAGTTGGTGAGATAAAAGCCCACCAAACGATGA<br>TACGTAGCGACCTGAGAGGGTAATCGGCCACATTGGGACTGAAAACGG<br>CCCAAACTCCTACGGGAGGCAGCAGTAGGGAATCTTCCACAATGGACG<br>AAAGTCTGATGAGCAACGCCGCGTGAGTGATGAAGGTTTTCGATCGTA<br>AAACTCTGTTGTTAAAGAAAAACAAGGTTAAAGTAACTGTTAACACTT<br>TGACGGTATTTAACCAAAAAGCCACGGCTAACTACGTGCCAGGCCGCG<br>GTAATACGTAGGGCAAGCGTTGTCCGGATTTATTGGGCGTAAAGCGAG<br>CGCAGGCGGTTTTGTAAGTCTGCTGTGAAAGCCCTCAGCTAACTGAGG<br>AAGTGCAGTGGAAACTACAAAACTTGAGTACAGAAGAGGAAAGTGGA<br>ACTCCATGTGTAGCGGTGAGTG | **KY027183.1** |

| | | | | | |
|---|---|---|---|---|---|
| k64 | mid gut | *Al. kunkeei* | 99.43 | AGTCGAACGAGCTCTCCCAAATTGATTTTATGCTTGCATAAATGATTTT<br>TGGATTCGGAGCGAGTGGCGAACTGGTGAGTAACACGTGGGTAACCTG<br>CCCCGAAGCGGGGGATAACATTTGGAAACAAGTGCTAATACCGCATAA<br>TTAGTTGGAACCGCATGGTTCCAATTTGAAAAAT | **MT381736.1** |
| k55 | mid gut | *Al. kunkeei* | 100 | AGTCGAACGAGCTCTCCCAAATTGATTTTATGCTTGCATAAATGATTTT<br>TGGATTCGGAGCGAGTGGCGAACTGGTGAGTAACACGTGGGTAACCTG<br>CCCCGAAGCGGGGGATAACATTTGGAAACAAGTGCTAATACCGCATAA<br>TTAGTTGGAACCGCATGGTTCCAACTTGAAAGATGGCTCTGCTATCACT<br>TTGGGATGGACCCGCGCCGTATTAG | **MT381736.1** |
| k40 | mid gut | *Al. kunkeei* | 100 | GTCGAACGAGCTCTCCCAAATTGATTTTATGCTTGCATAAATGATTTTT<br>GGATTCGGAGCGAGTGGCGAACTGGTGAGTAACACGTGGGTAACCTGC<br>CCCGAAGCGGGGGATAACATTTGGAAACAAGTGCTAATACCGCATAAT<br>TAGTTGGAACCGCATGGTTCCAACTTGAAAGATGGCTCT | **MT381736.1** |
| k89 | mid gut | *Al. kunkeei* | 100 | GTCGAACGAGCTCTCCCAAATTGATTTTATGCTTGCATAAATGATTTTT<br>GGATTCGGAGCGAGTGGCGAACTGGTGAGTAACACGTGGGTAACCTGC<br>CCCGAAGCGGGGGATAACATTTGGAAACAAGTGCTAATACCGCATAAT<br>TATTT | **MT381736.1** |
| k112 | mid gut | *Al. kunkeei* | 100 | CCAAATTGATTTTATGCTTGCATAAATGATTTTTGGATTCGGAGCGAGT<br>GGCGAACTGGTGAGTAACACGTGGGTAACCTGCCCCGAAGCGGGGGA<br>TAACATTTGGAAACAAGTGCTAATACCGCATAATTAGTTGGAACCGCA<br>TGGTTCCAACTTGAAAGATGGCTCTGCTATCACTTTGGGATGGACCCGC<br>GCCGTATTAG | **MT381736.1** |
| Lpla1 | mid gut | *Lp. plantarum* | 99.31 | AGTGGCGAACTGGTGAGTAACACGTGGGAAACCTGCCCAGAAGCGGG<br>GGATAACACCTGGAAACAGATGCTAATACCGCATAACAACTTGGACCG<br>CATGGTCCGAGTTTGAAAAATGGCTTCGGCTATCACTTTTGGATGGTCC<br>CGCGGCGTATTAGCTAGATGGGGGGGTAACGGCTCACCATGGCAATGA<br>TACGTAGCCGACCTGAGAGGGTAATCGGCCACATTGGGACTGAAACAC<br>GGCCCAAACTCCTACGGGAGGCAGCAGTAGGGAATCTTCCACAATGGA<br>CG | **KU892616.1** |
| k28 | honey stomach | *Al. kunkeei* | 100 | AAGTCGAACGAGCTCTCCCAAATTGATTTTATGCTTGCATAAATGATTT<br>TTGGATTCGGAGCGAGTGGCGAACTGGTGAGTAACACGTGGGTAACCT<br>GCCCCGAAGCGGGGGATAACATTTGGAAACAAGTGCTAATACCGCATA<br>ATTAGTTGGAACCGCATGGTTCCAACTTGAAAGATGGCT | **MT381736.1** |

| | | | | | |
|---|---|---|---|---|---|
| k50 | honey stomach | *Al. kunkeei* | 100 | CCAAATTGATTTTATGCTTGCATAAATGATTTTTGGATTCGGAGCGAGT<br>GGCGAACTGGTGAGTAACACGTGGGTAACCTGCCCCGAAGCGGGGGA<br>TAACATTTGGAAACAAGTGCTAATACCGCATAATTAGTTGGAACCGCA<br>TGGTTCCAACTTGAAAAAT | **MT381736.1** |
| k51 | honey stomach | *Al. kunkeei* | 100 | TCGAACGAGCTCTCCCAAATTGATTTTATGCTTGCATAAATGATTTTTG<br>GATTCGGAGCGAGTGGCGAACTGGTGAGTAACACGTGGGTAACCTGCC<br>CCGAAGCGGGGGATAACATTTGGAAACAAGTGCTAATACCGCATAATT<br>AG | **MT381736.1** |
| 84 | honey stomach | *Lb. lactis* | 96.19 | AGGTTGGTACTTGTACCGACTGGATGACACGAACGGGTGAGTAACGCG<br>GGGGGAATCTGCCTTTGAACGGGGGACAACATTTGGAAACCAATGCTA<br>ATACCGCG | **MT269313.1** |
| Ff36 | honey stomach | *F. fructosus* | 98.56 | GTGCTTGCACTTTCCAAGTAAGTGGCGAACGGGTGAGTAACACGTGAA<br>TAACCTACCTCAAAGTCTGGGATAACCATTGGAAACAGTGACTAATAC<br>CGGATAAAACCCAAGTGCACATGCACTAAGGTTAAAAGCTGCGTTTGC<br>AGCGCTTTAAAATGGATTCGCGGTGCATTATTTAGTTGGTGAGGTAAA<br>GGCTCACCAAGACGATGATGCATAGCCGAGTTGAGAGACTGACCGGCC<br>ACATTGGGACTGAAACACGGCCCAAACTCCTACGGGAGGCTGCAGTAG<br>GGAATCTTCCACAATGGGCGCAAGCCTGATGGAGCAACGCCGCGTGTG<br>TGATGAAGGCTTTCGGGTCGTAAAGCACTGTTGTATGGGAAAAACGGG<br>TTTAAAAGGAAATGCTTAAACAGTGACGGTACCATACCAAAAAGGGAC<br>GGCTAAATACGTGCCAGCAGCCGCGGTAATACGTATGTCCCGAGCGTT<br>ATCCGGATTTATTGGGCGTAAAGCGAGCGCAGACGGTTGCTTAAGTCT<br>GAAGTGAAAGCCCACAGCTTAACTGTGGAATGGCTTTGGAAACTGGGC<br>AACTTGAGTACAGTAGAGGTAAGTGGAACTCCATGTGTAGCGGTGAAT<br>GGCG | **MH796219.1** |
| Lpla67 | honey stomach | *Lp. plantarum* | 100 | ACTCTGGTATTGATTGGTGCTTGCATCATGATTTACATTTGAGTGAGTG<br>GCGAACTGGTGAGTAACACGTGGGAAACCTGCCCAGAAGCGGGGGAT<br>AACACCTGGAAACAGATGCTAATACCGCATAACAACTTGGACCGCATG<br>GTCCGAGTTTGAAAGATGGCTTCGGCTATCACTTTTGGATGGT | **MT645511.1** |
| Lpla6 | honey stomach | *Lp. plantarum* | 100 | GTGCTTGCATCATGATTTACATTTGAGTGAGGGGGGAACTGGTGAGTA<br>ACACGTGGGAAACCTGCCCAGAAGCGGGGGATAACACCTGGAAACAG<br>ATGCTAATACCGCATAACAACTTGGACCGCATGGTCCGAGCTTGAAAG<br>ATGGCTTCGGCTATCACTTTTGGATGGTCCCGCGGCGTATTAGCTAGAT<br>GGTGGGGTAACGGC | **LC159537.1** |

| | | | | | |
|---|---|---|---|---|---|
| Lpla62 | honey stomach | *Lp. plantarum* | 99.65 | GTTAAAGTCGAACGAACTCTGGTATTGATTGGTGCTTGCATCATGATTT<br>ACATTTGAGTGAGTGGCGAACTGGTGAGTAACACGTGGGAAACCTGCC<br>CAGAAGCGGGGGATAACACCTGGAAACAGATGCTAATACCGCATAAC<br>AACTTGGACCGCATGGTCCGAGTTTGAAAGATGGCTTCGGCTATCACTT<br>TTGGATGGTCCCGCGGCGTATTAGCTAGATGGTGGGGTAACGGCTCAC<br>CATGGCAATGATACGTACCCGACCTGAGAGGGTAATCGGCCACATTGG<br>GACTGAAACACGGCCCAAACTCCTACGGGAGGCAGCAGTAGGGAATC<br>TTCCACAATGGACGAAAGTCTGATGGAGCAACGCCGCGTGAGTGAAAA<br>AGGGTTTCGGCTCGTAAAACTCTGTTGTTAAAGAAAAACATATCTGAG<br>AGTAACTGTTCAGGTATTGACGGTATTTAACCAGAAAGCCACGGCTAA<br>CTACGTGCCAGCAGCCGCGGTAATACGTAGGTGGCAAGCGTTGTCCGG<br>ATTTATTGGGCGTAAAGCGAGCGCAGGCGGTTTTTTAAGTCTGATG | **MT184183.1** |
| Lpla8 | honey stomach | *Lp. plantarum* | 100 | TAAAGTCGAACGAACTCTGGTATTGATTGGGGCTTGCATCATGATTTAC<br>ATTTGAGTGAGTGGCGAACTGGTGAGTAACACGTGGGAAACCTGCCCA<br>AAAGCGGGGGATAACACCTGGAAACAGATGCTAATACCGCATAACAA<br>CTTGGACCGCATGGTCCGAGTTTGAAAGATGGCTTCGGCTATCACTTTT<br>GGATGGTCCCGCGGCGTATTAGCTAAATGGGGGGGTAACGGCTCACCA<br>TGGCAATGATACGTACCCGACCTGAGAGGGTAATCGGCCACATTGGGA<br>CTGAAACACGGCCCAAACTCCTACGGGAGGCAGCAGTAGGGAATCTTC<br>CACAATGGACAAAAGTCTGATGGAGCAACGCCGC | **MH392856.1** |
| Lpla10 | honey stomach | *Lp. plantarum* | 100 | TAAAGTCGAACGAACTCTGGTATTGATTGGTGCTTGCATCATGATTTAC<br>ATTTGAGTGAGTGGCGAACTGGTGAGTAACACGTGGGAAACCTGCCCA<br>GAAGCGGGGGATAACACCTGGAAACAGATGCTAATACCGCATAACAA<br>CTTGGACCGCATGGTCCAAGTTTGAAAGATGGCTTCGGCTATCACTTTT<br>GGATGGTCCCGCGGCGTATTAGCTAGATGGGGGGGTAACGGCTCACCA<br>TGGCAATGATACGTACCCGACCTGAGAGGGTAATCGGCCACATTGGGA<br>CTGAAACACGGCCCAAACTCCTACGGGAGGCAGCAGTAGGGAATCTTC<br>CACAATGGACGAAAGTCTGATGGAGCAACGCCGCGTGAGTGAAAAAG<br>GGTTTCGGCTCGTAAAACTCTGTTGTTAAAGAAAAACATATCTGAGAG<br>TAACTGTTCAGGTATTGACGGTATTTAACCAGAAAGCCACGGCTAACT<br>ACGTGCCAGCAGCCGCGGTAATACGTAGGTGGCAAGCGTTGTCCGGAT<br>TTATTGGGCGTAAAGCGAGCGCAGGCGGTTTTTTAAGTCTGATGTGAA<br>AGCCTTCGGCTCAACCGAAAG | **MT184183.1** |

| | | | | | |
|---|---|---|---|---|---|
| Lpla12 | honey stomach | *Lp. plantarum* | 98.47 | TAAAAAGTCGAACGAACTCTGGTATTGATTGGGGCTTGCATCATGATTTACATTTGAGTGAGTGGCGAACTGGTGAGTAACACGTGGGAAACCTGCCCAAAAGCGGGGGATAACACCTGGAAACAGATGCTAATACCGCATAACAACTTGGACCGCATGGTCCAAGTTTGAAAGATGGCTTCGGCTATCACTTTTGGATGGTCCCGCGGCGTATTAGCTAGATGGGGGGGTAACGGCCACCATGGCAATGATACGTAGCCGACCTGAGAGGGTAATCGGCCACATTGGGACTGAAACACGGCCCAAACTCCTACGGGAGGCAGCAGTAGGGAATCTTCCACAATGGACAAAAGTCTGATGGAGCAACGCCGCGTGAGTGAAAAAGGGTTTCGGC | **CP055123.1** |
| Lpla13 | honey stomach | *Lp. plantarum* | 98.53 | CGAACTCTGGTATTGATTGGTGCTTGCATCATGATTTACATTTGAGTGAGTGGCGAACTGGTGATAA | **MT645511.1** |
| Lpla14 | honey stomach | *Lp. plantarum* | 100 | ACGAACTCTGGTATTGATTGGTGCTTGCATCATGATTTACATTTGAGTGAGGGGCGAACTGGTGATA | **LC159530.1** |
| Lpla72 | honey stomach | *Lp. plantarum* | 100 | CGAACTCTGGTATTGATTGGGGCTTGCATCATGATTTACATTTGAGTGAGTGGCGAACTGGTGATAACACGTGGGAAACCTGCCCAAAAGCGGGGGATAACACCTGGAAACAGATGCTA | **JX003603.1** |
| Lpla24 | honey stomach | *Lp. plantarum* | 100 | CGAACTCTGGTATTGATTGGGGCTTGCATCATGATTTACATTTGAGTGAGTGGCGAACTGGTGATAACACGTGGGAAACCTGCCCAAAAGCGGGGGATAACACCTGGAAACAGATGCTA | **JX003603.1** |

*Accession number of the sequence of the closest relative found by blast search
